# MEGAnnotator2: a pipeline for the assembly and annotation of microbial genomes

**DOI:** 10.20517/mrr.2022.21

**Published:** 2023-04-30

**Authors:** Gabriele Andrea Lugli, Federico Fontana, Chiara Tarracchini, Christian Milani, Leonardo Mancabelli, Francesca Turroni, Marco Ventura

**Affiliations:** ^1^Laboratory of Probiogenomics, Department of Chemistry, Life Sciences, and Environmental Sustainability, University of Parma, Parma 43124, Italy.; ^2^Microbiome Research Hub, University of Parma, Parma 43124, Italy.; ^3^Department of Medicine and Surgery, University of Parma, Parma 43125, Italy.

**Keywords:** Genomics, bioinformatics, next-generation sequencing

## Abstract

The reconstruction of microbial genome sequences by bioinformatic pipelines and the consequent functional annotation of their genes’ repertoire are fundamental activities aiming at unveiling their biological mechanisms, such as metabolism, virulence factors, and antimicrobial resistances. Here, we describe the development of the MEGAnnotator2 pipeline able to manage all next-generation sequencing methodologies producing short- and long-read DNA sequences. Starting from raw sequencing data, the updated pipeline can manage multiple analyses leading to the assembly of high-quality genome sequences and the functional classification of their genetic repertoire, providing the user with a useful report constituting features and statistics related to the microbial genome. The updated pipeline is fully automated from the installation to the delivery of the output, thus requiring minimal bioinformatics knowledge to be executed.

## INTRODUCTION

Since 1995, whole genome sequencing (WGS) has been the golden standard for the reconstruction of microbial genome sequences, with the publication of the first complete genome sequence of *Haemophilus influenza*^[1]^. WGS was an efficient strategy that allowed gathering random DNA sequences of a microbial genome used to reconstruct the entire chromosome sequence using mathematical algorithms^[2]^. Nowadays, the most common DNA sequencing technologies used for the reconstruction of genomes are represented by Illumina, followed by Pacific Bioscience and Oxford Nanopore^[3,4]^. While the first one is largely used for the ability to produce a massive amount of high-quality data, it relies on the production of short DNA sequences ranging from 150 to 250 bp^[5]^. Instead, PacBio and Nanopore sequencing systems are technologies chosen for the genome reconstruction of microorganisms thanks to their ability to produce long DNA sequences up to 40,000 bp^[6]^. However, the latter technologies, also known as third-generation sequencers, display some limitations in accuracy and throughput with respect to short-read sequencing. Nonetheless, the advent of long-read DNA sequencers allowed to improve draft assembly of microbial genomes, producing complete genome sequences^[7]^, and, recently, the implementation of PacBio HiFi reads drastically improved the long-read DNA final quality.

Accordingly, sequenced genomic data needs to be processed by bioinformatic tools to reconstruct the chromosomal sequences and unveil their genomic repertoire^[8,9]^. Thus, software for assembling and annotating microbial genomes has been implemented to process and manage such DNA data^[10-13]^. In 2016, the MEGAnnotator pipeline was implemented to provide the researcher with automated *in silico* tools for analyzing prokaryotic genomes^[14]^. Nowadays, many pipelines have been implemented to ease genomes assembly and annotation process^[15,16]^. Nevertheless, selecting free software that manages all types of sequenced DNA to be used in a local environment is still highly challenging.

Here, we describe the improved bioinformatic pipeline MEGAnnotator2 that allows the assembly of prokaryotic genomes and chromosomes from unicellular eukaryotes, followed by gene prediction, functional annotation, and DNA quality evaluation of the reconstructed genome sequences. The pipeline can manage data from every NGS platform and modern third-generation sequencers such as PacBio and Nanopore long reads. Furthermore, each analysis step is automated and managed by a bash script, which coordinates freely online available software and custom databases that are continuously kept updated to overcome issues related to taxonomy re-classification.

## MATERIALS AND METHODS

### MEGAnnotator2 workflow

**Figure 1:**
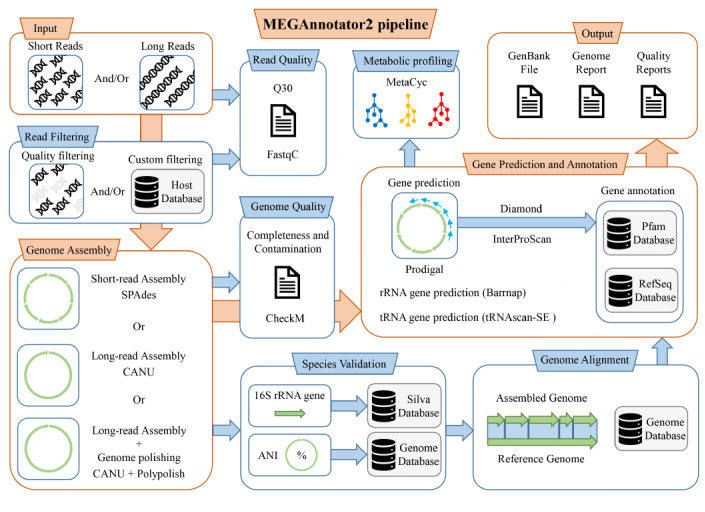
Schematic representation of the workflow. Starting from raw reads obtained from NGS platforms, MEGAnnotator2 will perform read filtering, assembly, quality control of genome sequences, genome alignments, genome comparisons, gene prediction, gene annotation, and metabolic profiling. Red arrows highlight the mandatory steps of the pipeline.

MEGAnnotator2 is a bash script that runs on Linux under GNU General Public License (GPL). The complete workflow reported in Figure 1 shows the different steps managed by the pipeline by relying on the coordination of freely available software programs. Complete execution of the pipeline starts from the filtering of the raw sequencing data, providing statistics on the quality of the sequenced DNA as well as the filtered DNA that will be used for the assembly of the microbial genome. Based on the sequencing technology (short reads, long reads, or both), a specific assembly strategy is employed, resulting in one or more consensus sequences of the microbial chromosomes. Then, a quality assessment of the assembled data is performed to highlight the genome quality and the species relatedness. The latter information will be used to reorder contigs based on the reference strain of the identified species. Later, the pipeline proceeds with the prediction of the coding genes (as well as non-coding genes) to predict their function using similarity searches in the custom NCBI RefSeq database and a domain search in the InterProScan database. Gathered data will be used to generate a GenBank file that stores all biological information while all main statistics are reported in an available text file. Finally, the pipeline performs a metabolic screening to retrieve each attributable enzymatic reaction to predicted genes.

As the previous version of the pipeline, MEGAnnotator2 aims to improve every step described above to enhance the quality of genome sequences and gene annotation by reducing the time and effort required, thanks to its automated execution. In fact, the user needs only to provide the individual NGS data, and the MEGAnnotator2 pipeline will manage all steps, ultimately leading to the generation of a GenBank file, thus leaving the user free to carry out other activities. To enhance the performance of each software package, MEGAnnotator2 handles the execution of multiple threads as set up in the MEGAnnotator2 parameters. Accordingly, software parameters can be personalized in the parameter file of MEGAnnotator2 to guide the execution of each software package by the pipeline without the need to set up the individual programs. The modular implementation of the pipeline grants flexible execution of the analyses, allowing the user to select which step to perform and allowing modifying parameters based on the user’s need. For a detailed overview of the pipeline’s editable parameters, refer to the manual.

The previous version of the software was distributed with distinct virtual machines containing different databases for gene annotation since many dependencies needed to be installed in the user environment. Instead, MEGAnnotator2 is provided by an auto installer able to automatically manage the installation of dependencies, the set-up of the software, and the download of pre-processed databases whose size has been significantly reduced. The installer file of MEGAnnotator2 can be downloaded at the link http://probiogenomics.unipr.it/cmu/. As reported in the manual, a single Unix command line is needed to have the full pipeline installed in the system. One of the advantages of using MEGAnnotator2 is that it can be used without internet access since all the programs and databases will be accessible locally after the pipeline installation.

Another main novelty in pipeline execution is the possibility of processing multiple genomes in series without wasting time between analysis execution. Specifically, the script can recognize multiple FASTQ files retrieved from NGS base-calling and organize the execution in tandem with the analysis based on the parameters arranged by the user. Furthermore, the results of multiple analyses can be put together to provide an overall view of the assembled data. In MEGAnnotator2, the implementation of the automated script is easy to achieve, as reported in the manual. Thus, additional extensions will be implemented in future updates of the software and it can also be programmed and introduced by the user base on the need.

### MEGAnnotator2 databases

Alongside the software, MEGAnnotator2 is provided with multiple databases to avoid restricted online computing during the execution of the pipeline. Specifically, alongside the installation and software update, the pipeline can be run on a local machine without constant network access. Notably, four *ad-hoc* pre-compilated databases are downloaded together with all the scripts to use the pipeline at its full potential.

The first database is dedicated to the functional annotation of genes, aiming at providing reliable outputs with the most up-to-date data for gene classification. To do so, the RefSeq database of NBCI (amino acid sequences) is processed by removing non-informative genes, such as hypothetical proteins, and a collection of inappropriate gene names that may compromise the feasibility of the resulting functional classification. Then, selected genes are clustered with CD-HIT using a sequence identity threshold of 70%^[17]^. This process reduces the overall size of the database without removing any sequence information, resulting in a decreased computational cost for the system of the final user. Using this strategy, we reduced the previous database of MEGAnnotator from hundreds of gigabytes to 35 gigabytes. However, as well as for the other databases provided by MEGAnnotator2, the installation of the software will download the pre-processed database. Thus, the user does not need to process or compile individual databases.

A second database is represented by a single reference genome for each species of microorganism, covering all known genome variability but avoiding redundancy within the same species. All bacterial genomes available in the NCBI RefSeq database were retrieved and filtered based on the most up-to-date reference ANI table made available from the repository. Finally, for each bacterial species, each genome was processed using the sourmash software^[18]^ and compared in a pair-wise approach to obtain a series of Jaccard similarity matrices. Then, the optimal reference genome was extracted from each Jaccard similarity matrix, given by the highest average Jaccard similarity score. Genome sequences of representative genomes are used to provide average nucleotide identity (ANI) values with respect to the assembled genome sequence. Furthermore, a subset of the database, represented by complete reference genome only, is used to perform sequence alignment, allowing contig reordering of partially reconstructed chromosomal sequences.

The third database represents a collection of validated 16S and 18S rRNA gene sequences of all classified microorganisms based on the SILVA repository^[19]^. Specifically, the database is generated by processing the latest release of the complete SILVA repository removing sequences with non-informative microbial taxonomy, such as unknown species. Then, selected ribosomal genes are clustered with CD-HIT using a sequence identity threshold of 99.9%^[17]^. At first glance, results based on this database might appear redundant since, through the MEGAnnotator2 pipeline, the species is attributed through ANI values comparison. The issue is that, to date, we do not possess the genome sequence of all known microorganisms. Thus, additional information, such as sequence similarity of the 16S/18S rRNA gene, can be helpful in studying uncommon microorganisms.

Finally, MEGAnnotator2 is provided with a database comprising information regarding metabolic reactions collected from the MetaCyc^[20]^. By using the latter database, it is possible to have a profile constituting each attributable enzymatic reaction of the predicted microorganism genes in analysis.

All databases will be updated every six months to overcome taxonomy re-classification issues and provide reliable output profiles. The support will end when updated methodologies overcome the current classification strategies, resulting in a reshaping of the pipeline and databases. The user can also provide custom databases to perform custom DNA filtering steps before assembly. These additional databases need to be compiled using the BWA aligner^[21]^ as reported in the manual, starting from DNA sequences in fasta format.

### MEGAnnotator2 input files

To run MEGAnnotator2, the user needs to provide DNA sequencing data in fastq format. Short reads in single- or paired-end can be used (Illumina or Ion Torrent data) as well as long reads (PacBio or Nanopore data). In this context, PacBio HiFi reads can only be used prior to conversion from BAM to fastq format. The pipeline can be executed in a Unix terminal with a single command, specifying the name of the project and the input data path. For example, it follows three commands based on paired-end, long reads, and mixed reads input:

MEGAnnotator2 -t 60 -n project_name -p -f forward_input.fastq -r reverse_input.fastq

MEGAnnotator2 -t 60 -n project_name -l -i input.fastq

MEGAnnotator2 -t 60 -n project_name -o -i long_input.fastq -f forward_input.fastq -r reverse_input.fastq

Otherwise, dedicated scripts are implemented in MEGAnnotator2 to automatize the processing of the input data generating a bash script that will run samples in series without the need to execute specific commands. For additional information on the execution of the program, see the manual.

#### Step 1: quality filtering of the data

To provide more reliable results, we implemented a DNA filtering step, a feature absent in the previous version of MEGAnnotator^[14]^. As default, MEGAnnotator2 performs a quality filtering step aiming at removing DNA sequences that are too short or that display low quality. Based on the input file typology, the pipeline will perform a short read filtering (single or paired-end based on the technology) or a long read filtering of the data. To do so, the fastq-mcf utility (https://github.com/ExpressionAnalysis/ea-utils) is employed to perform filtering of short reads, removing as default reads shorter than 100 nucleotides and those with a quality < 20. Otherwise, long reads were managed by Fitlong (https://github.com/rrwick/Filtlong), removing as default reads shorter than 1,000 nucleotides and keeping 90% of reads with superior quality not exceeding 500 Gb of data. Whenever both short and long reads data are used as input, Fitlong will better evaluate the long read quality using k-mer matches to the short read to improve the final genome quality. The user can manually edit all parameters to achieve a more suitable filtering step based on the user’s needs.

Furthermore, the pipeline allows a custom filtering step before assembly to remove putative contamination that may occur in strain isolation or sequencing procedure. For example, the user may choose to remove the DNA of a specific bacterial species or DNA vector sequences used in certain experimental procedures.

Moreover, as a new feature, the pipeline generates statistics for each fastq input file to certify its quality. More in specific, a pre-filtering and post-filtering analysis is managed by the FastQC quality control tool to spot potential problems in the sequencing dataset used. Data regarding base quality scores, read quality scores, sequence length distribution, sequence duplication levels, and overrepresented sequences are displayed before and after read filtering.

#### Step 2: assembly of the filtered reads

After a first quality filtering of the input data, assemblies of DNA sequences can be performed using a combination of short and long sequences obtained by any NGS platform as well as modern third-generation sequencers such as PacBio and Nanopore. Filtered short reads are managed by SPAdes^[22]^, which evaluates the average length of the DNA sequences to generate an optimum list of k-mer sizes to be used as a parameter in the assembly phase. For example, an Illumina 250bp paired-end output will result in a list of “21,33,55,77,99,127” k-mer sizes. Besides, the assembler CANU manages long-read sequences^[23]^. To obtain more reliable data, which usually consists of a complete reconstruction of the chromosomal sequence, the user can input the putative length of the genome sequence to the pipeline, which will be used as a variable in the assembly step.

Furthermore, the pipeline can also manage assemblies using short and long-read sequences as input. MEGAnnotator2 gives the user the possibility to choose between two strategies. The first approach takes advantage of the capability of SPAdes to manage hybrid assemblies. Thus, the assembled chromosomal sequence obtained from a long-read assembly managed by CANU is then used as input by SPAdes as a reference to perform the hybrid assembly together with long and short-read sequences. Otherwise, the second approach uses once again the assembled chromosomal sequence obtained from the long read assembly, followed by DNA sequence polishing using the Polypolish tool^[24]^. The resulting high-quality genome is obtained by aligning each short read to all possible locations of the assembled genome by making use of the SAM file generated by the BWA aligner^[21]^. Both methodologies can be used to generate a high-quality complete genome sequence of the assembled genomes. Nonetheless, based on our validation test, the polishing approach can minimize INDELs’ occurrence in the genome sequence.

#### Step 3: genome quality check (optional)

As a new feature of MEGAnnotator2, assembled data is assessed with multiple validation methods. A first screening is represented by the identification of the assembled genomes of the microbial species. The 16S/18S rRNA gene sequences are compared to the non-redundant SILVA database above described through BLASTn^[25]^. At the same time, the fastANI tool is used to identify the microorganism with the highest whole-genome Average Nucleotide Identity (ANI) values^[26]^. Together, those microorganisms with the highest 16S/18S rRNA gene sequence identity and highest ANI values, composed with the respective values, are reported as genome information in the output.

The average genome coverage is calculated using the BBmap aligner (https://github.com/BioInfoTools/BBMap) by mapping the short reads on the assembled contig sequences. Instead, the coverage of long-read assemblies is retrieved directly from the CANU report. Additionally, the quality of the assembled genome is evaluated using the checkM tool^[27]^. Data regarding the completeness and contamination of the reconstructed genome are reported as values in the output information.

As in the first MEGAnnotator version, resulting contig sequences retrieved from the assemblies can be reordered based on a reference genome sequence of the same species. The difference in this software version is that the user does not need to provide the genome sequence of the reference strain, but only the species name in the parameters file. Doing that, the pipeline will retrieve the genome sequence of the reference strain from the RefSeq genomes of NCBI and provide to perform a genome alignment using MAUVE^[28]^. If the ANI analysis results are discordant with the given species name, MEGAnnotator2 will choose the appropriate genome for the reordering.

Finally, assembled contigs are filtered based on length before gene prediction and functional annotation. The user can provide two different length cut-offs to remove contigs with an inferior length obtained through the short read assembly (using SPAdes) or long read assembly (using CANU).

#### Step 4: gene prediction and functional annotation

Gene prediction is performed by prodigal^[29]^, whose high efficiency in predicting the start of genes has been documented^[30]^. Collected amino acid gene sequences are then used to perform their functional prediction. Notably, partial sequences predicted at the edge of contigs (genes without the start and/or the stop codon) may be removed based on their length by the user. The functional annotation of each gene sequence is managed by DIAMOND, due to its reduced computational run time with respect to other similar tools^[31]^. By default, DIAMOND performs alignment using the --sensitive option in search of query coverage > 50 and e-value < 1·10^-8^. However, like the other parameters described above, they can be easily customized by modifying their values in the parameters file. Thus, the putative function of the subject sequence with the highest score is attributed to each query sequence.

Unclassified genes from the DIAMOND search are further investigated by InterProScan among an HMM-based database^[32]^, aiming at classifying them into family proteins and predicting domains that may suggest their biological role. If a gene is unclassified even in the InterProScan profiling, the resulting functional annotation is set as a “hypothetical protein”.

Additionally, non-coding genes are predicted using barrnap (https://github.com/tseemann/barrnap) and tRNAscan-SE 2.0^[33]^, allowing for detecting rRNA and tRNA genes across the assembled genome sequence. In this regard, the pipeline can be programmed to process prokaryotes or eukaryotes genomes to predict the appropriate ribosomal genes using the -k (kingdom) option or setting the parameter file. By default, MEGAnnotator2 will predict ribosomal genes associated with prokaryotes.

#### Step 5: metabolic profiling (optional)

As a new feature of MEGAnnotator2, predicted gene sequences are screened against the MetaCyc metabolic database to retrieve each attributable enzymatic reaction^[20]^. The Enzyme Commission (EC) numbers are conferred to each amino acid sequence using DIAMOND^[31]^. By default, DIAMOND performs alignment using the --sensitive option in search of query coverage > 50 and e-value < 1·10^-8^. Results of the analysis are reported as raw counts for each EC number as well as a percentage based on the total number of genes.

### MEGAnnotator2 output files

The amount of output files provided by MEGAnnotator2 is proportional to the number of analyses defined in the parameters file. By default, the process of assembly and annotation of the microbial genomes ends with the generation of a GenBank file compatible with the Artemis genome browser^[34]^. Within the GenBank file is reported information about the genome sequence, gene positions, and gene annotation. Furthermore, a comprehensive file (genome_info.txt) reports the main characteristics of the assembled microbial genomes, including the amount of the DNA sequencing output, number of filtered reads, number of assembled contigs, genome length, average coverage, completeness of the genome and its contamination level, number of genes, rRNA genes, and tRNA genes, and species prediction based on the 16S/18S rRNA gene sequence and ANI values of the chromosomal sequence.

Additional files are produced to allow the user to evaluate the results of each step of the pipeline. Among these files is reported the quality of the genome sequence (checkM_report), the results of the 16S/18S rRNA gene alignment (16S.blastn or 18S.blastn), the collection of gene protein sequences (aaORFs.fasta), the sequence of the assembled contigs (contigs.fasta) and a report of long read sequence polishing if requested (polishing_report.txt).

In addition, multiple folders are provided, containing data regarding the main steps of genome processing. Filtered reads are stored as FASTQ files in the folder “filtered_reads” together with html files reporting the quality of raw reads and filtered reads if requested. Genome alignment of the assembled data with respect to the reference genome retrieved from the ANI database is located in the folder “mauve_alignment” and can be visualized by using MAUVE. Furthermore, the assembly documentation produced by SPAdes or CANU is located in the “assembly” folder, including statistics, assembly steps, and logs. Finally, the folder named “metabolic_reactions” contains the results achieved from the metabolic profiling if requested by the user.

In case multiple microbial strains have been analyzed in tandem with MEGAnnotator2, multiple folders will be generated for each analyzed genome named with the microorganism code (project_name).

## RESULTS AND DISCUSSION

### MEGAnnotator2 performance and statistics using short reads

This work aims to deliver a complete pipeline to manage any sequencing output and provide the user with statistics and biological information about the assembled microorganism. Thus, each available online software included in the pipeline has been chosen based on recent scientific literature highlighting its performance with respect to other tools^[30,31,35]^.

**Figure 2:**
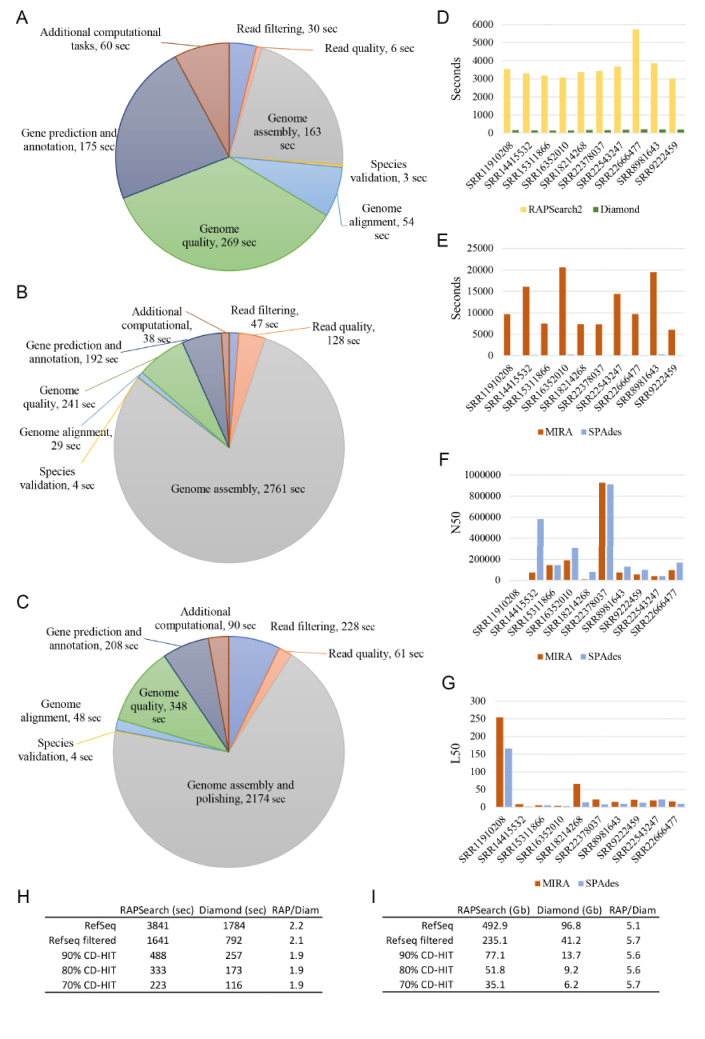
MEGAnnotator2 execution time of each task and comparison with the old pipeline. Panel (A) shows the computational time of each step of the pipeline based on the performance observed through processing ten microbial genomes sequenced with short-read technologies (1,000,000 reads). Panel (B) exhibits the same data based on long reads input (500,000 reads), while panel (C) displays the computational time using a hybrid approach involving both short- and long-reads data. Panels (D) and (E) denote the computational time of annotation and assembly software, respectively. Panels (F) and (G) show the N50 and L50 of each assembly, respectively. Finally, panels (H) and (I) display the difference in time and space of different clustering of the RefSeq databases with CD-HIT.

To test the whole pipeline, we used one million short reads belonging to 10 microbial species characterized by different genome sizes, ranging from two Mb to five Mb [Table 1]. The machine used to benchmark the pipeline was equipped with an AMD Threadripper with 32 cores and 256 GB of RAM. Memory read and write operations were managed by an NVME m.2 2tb SSD. The average execution time of the complete pipeline was 14.2 min, while mandatory steps (assembly and annotation) took an average of 5.6 min to be executed. Figure 2 reported the individual timing of each step, with the genome quality step representing the most time-consuming (median of 269 sec), followed by the gene prediction and annotation (median of 175 sec) and the genome assembly (median of 163 sec) [Figure 2A and Supplementary Table 1]. An example of relevant statistics provided by MEGAnnotator2 to the user is reported in Table 1 and can be found as results once the pipeline has ended its job as a text document.

**Table 1:** MEGAnnotator2 report of 10 sequentially processed microbial genomes using short-read technology

**SRA**	**Sequencing output**	**High quality reads**	**Filtered reads**	**16S rRNA gene identity**	**ANI screening**	**Genome completeness**	**Genome contamination**	**Average coverage**	**Number of contigs**	**Genome length**	**Number of genes**	**Number of rRNA genes**	**Number of tRNA genes**
SRR11910208	1000000	630615	630615	*Streptococcus salivarius* subsp. *thermophilus* 100%	*Streptococcus thermophilus* 99.2%	99.89%	11.19%	51	913	2,904,834	2,934	7	50
SRR14415532	1000000	998717	998716	*Leuconostoc mesenteroides* 100%	*Leuconostoc suionicum* 93.9%	100%	0.18%	154	13	2,110,850	2,089	5	55
SRR15311866	1000000	999947	999947	*Bifidobacterium breve* JCM 7019 99.7%	*Bifidobacterium breve* 98.3%	100%	0.12%	126	35	2,374,842	2,011	3	55
SRR16352010	1000000	997594	996662	*Bifidobacterium longum* 100%	*Bifidobacterium longum* 98.7%	100%	0%	254	17	2,365,405	1,959	3	57
SRR18214268	1000000	726379	726379	*Lactobacillus paracasei* 100%	*Lacticaseibacillus paracasei* 99.0%	99.46%	0%	67	90	3,055,144	2,903	5	54
SRR22378037	1000000	892220	892220	*Lactococcus lactis* 99.9%	*Lactococcus cremoris* 88.0%	100%	0%	72	63	2,460,545	2,462	4	58
SRR22543247	1000000	998973	998973	*Enterococcus faecium* 100%	*Enterococcus faecium* 94.6%	99.63%	0.50%	104	147	3,1005,07	3,022	8	59
SRR22666477	1000000	986064	986062	*Shigella sonnei* 99.9%	*Shigella boydii* 98.7%	99.93%	0.33%	58	76	5,089,127	4,834	9	86
SRR8981643	1000000	997089	997089	*Clostridium botulinum* 100%	*Clostridium cagae* 97.6%	100%	0%	140	47	3,825,030	3,529	14	77
SRR9222459	1000000	841930	841930	*Faecalibacterium prausnitzii* 99.9%	*Faecalibacterium duncaniae* 85.8%	100%	0.14%	72	87	3,356,538	3,213	9	63

### Performance and statistics of the pipeline using long reads

Unlike short read analyses, the usage of long read sequences resulted in a more time-consuming procedure due to the implementation of dedicated filtering and assembly algorithms. To benchmark the efficiency of MEGAnnotator2 using long reads, additional 10 microbial strains were subjected to genome assembly and annotation using long reads, and additional 10 microbial strains with a combination of long and short reads [Tables 2 and 3]. Notably, aiming at simulating a real-world scenario, the microorganisms’ genome length used in the hybrid approach was larger than five Mb except for one [Table 3]. Testing has been performed using 500,000 long reads coupled with one million short reads for the hybrid approach. The average execution of the complete pipeline using long reads was 56.5 min, with the assembly step managed by CANU representing the most time-consuming (median of 2,761 sec) [Figure 2B]. Instead, by using a combination of different sequences, MEGAnnotator2 takes an average of 53.5 min, validating the assembly step of long reads to be the most complex procedure to date [Figure 2C]. Furthermore, using a hybrid approach, we highlighted the impact of long read filtering using the information of short reads that takes approximately five times more than the long read filtering alone, while the polishing of the assembled data takes additional 3.4 min [Supplementary Tables 2 and 3].

**Table 2:** MEGAnnotator2 report of 10 sequentially processed microbial genomes using long-read technology

**SRA**	**Sequencing output:**	**High quality reads:**	**16S rRNA gene identity:**	**ANI screening:**	**Genome completeness:**	**Genome contamination:**	**Average coverage:**	**Number of contigs:**	**Genome length:**	**Number of genes:**	**Number of rRNA genes:**	**Number of tRNA genes:**
SRR12201911	500000	59283	*Leuconostoc suionicum* 99.4%	*Leuconostoc mesenteroides* 96.7%	98.41%	3.45%	148	71	2,724,779	2,995	21	86
SRR13648750	500000	47731	*Lactococcus lactis* 99.9%	*Lactococcus lactis* 88.1%	100%	3.83%	154	9	2,581,970	2,547	19	65
SRR15521836	500000	127174	*Bacteroides salyersiae* 99.9%	*Bacteroides salyersiae* 99.0%	97.63%	1.49%	78	18	5,430,481	4,655	15	77
SRR17126341	500000	48110	*Eubacterium eligens* 100%	*Eubacterium eligens* 100%	98.25%	8.13%	153	5	2,963,578	2,611	15	47
SRR17126949	500000	49224	*Prevotella copri* 99.8%	*Prevotella copri* 100%	97.97%	2.36%	114	11	3,688,102	3,133	20	63
SRR17873544	500000	86673	*Clostridium innocuum* 99.9%	*Clostridium innocuum* 97.1%	100%	1.42%	53	14	5,032,638	4,937	12	48
SRR17873548	500000	119899	*Enterococcus hirae* 99.9%	*Enterococcus hirae* 98.9%	96.16%	2.90%	76	89	3,181,147	3,053	23	84
SRR21075862	500000	45558	*Streptococcus salivarius* subsp. *thermophilus* 99.9%	*Streptococcus thermophilus* 99.2%	99.89%	4.44%	176	4	1,910,856	2,016	18	67
SRR21276823	500000	49609	*Bifidobacterium animalis* subsp. *lactis* 100%	*Bifidobacterium animalis* 95.7%	100%	7.33%	180	6	2,041,333	1,653	9	58
SRR22159808	500000	49946	*Klebsiella pneumoniae* 100%	*Klebsiella pneumoniae* 99.0%	99.40%	0.13%	83	10	5,635,075	5,295	25	91

**Table 3:** MEGAnnotator2 report of 10 sequentially processed microbial genomes using short- and long-read technologies

**SRA**	**Sequencing output:**	**High quality reads:**	**Sequencing output (long reads):**	**High quality reads (long reads):**	**16S rRNA gene identity:**	**ANI screening:**	**Genome completeness:**	**Genome contamination:**	**Average coverage:**	**Average coverage (long reads):**	**Number of contigs:**	**Genome length:**	**Number of genes:**	**Number of rRNA genes:**	**Number of tRNA genes:**
ERR5950940	1000000	973651	73857	42648	*Shigella boydii* 99.9%	*Shigella boydii* 98.2%	99.97%	0.75%	75	39	55	6,606,912	6,860	22	97
ERR5951433	559060	514029	198629	60015	*Klebsiella pneumoniae* 99.9%	*Klebsiella pneumoniae* 99.0%	100%	1.04%	45	86	4	5,595,850	5,217	25	88
SRR12326962	369346	357819	92666	62330	*Salmonella enterica* 100%	*Salmonella enterica* 98.8%	100%	0.78%	32	97	5	5,310,747	5,134	22	92
SRR12811523	1000000	991228	196879	106628	*Enterobacter aerogenes* 99.9%	*Klebsiella aerogenes* 95.8%	100%	1.11%	49	99	8	5,567,807	5,281	25	90
SRR12811532	1000000	995953	102851	77655	*Citrobacter freundii* 100%	*Citrobacter portucalensis* 94.7%	99.94%	1.86%	48	99	16	5,693,281	5,598	25	86
SRR12811547	1000000	996250	222219	79032	*Enterobacter cloacae* 100%	*Enterobacter cloacae* 99.7%	98.92%	1.06%	50	98	19	5,496,621	5,248	25	87
SRR13177364	1000000	962158304	158304	61251	*Kocuria varians* 100%	n.d.	98.68%	0%	163	120	2	2,848,142	2,432	9	48
SRR13249533	850449	845436	109409	43592	*Bacillus oleronius* 99.8%	*Heyndrickxia oleronia* 100%	98.30%	6.27%	76	68	2	5,364,016	5,378	36	145
SRR14087463	1000000	991529	90337	62461	*Pseudomonas aeruginosa* 100%	*Pseudomonas aeruginosa* 94.1%	99.60%	0.17%	41	66	14	6,729,107	6,213	12	68
SRR21755520	1000000	944753	137078	25119	*Agrobacterium arsenijevicii* 98.9%	n.d.	97.26%	3.31%	46	52	4	5,773,279	5,401	15	60

Thus, based on the achieved results, MEGAnnotator2 can manage all its functions in approximately 14.5 min for short reads, 56.5 min for long reads, and 53.5 min using hybrid reads. Even if the hybrid pipeline introduces two additional analyses represented by long-read filtering by short-read data and genome sequence polishing, the average computing time of the pipeline is the same, highlighting high variability in the capability of the assembler to manage long reads data. If the user is not interested in statistics, essential functions will take approximately 5.6 min for short reads, 49.2 min for long reads, and 40 min for the usage of hybrid reads. Furthermore, no additional time is spent between analyses using the additional function of MEGAnnotator2 to manage multiple samples.

### MEGAnnotator2 improvement with respect to the old version

To highlight the enhancement made in MEGAnnotator2, a comparison against the first version (MEGAnnotator) was performed. Several features of the updated pipeline were not compared due to their absence in MEGAnnotator, *e.g.*, quality reports of sequenced reads, genome quality assessments, and metabolic profiling. Furthermore, since the older pipeline version cannot manage long reads data, we only employed the short reads belonging to the 10 microbial species above used to validate the new pipeline version [Table 1]. An updated RefSeq database was downloaded from NCBI and formatted using Rapsearch2 as reported in the MEGAnnotator manual to compare the pipeline efficiency. Furthermore, the last version of the MIRA assembler has been installed on the same machine used for MEGAnnotator2 benchmarking.

Focusing on the assembled genomes, we observed that MEGAnnotator generates a higher number of contigs with respect to the oldest version [Figure 2]. Moreover, the assembled genomes of the oldest version of the pipeline were characterized by a lower number of N50 and higher number of L50, *i.e.*, 74,157 and 18, in respect to the updated pipeline, *i.e.*, 137,143 and 10 [Figure 2F]. Furthermore, in the previous software version, the user was forced to provide the reference genome sequence in the same analysis folder. Thus, MEGAnnotator2 can assemble microbial genomes more efficiently, and the selection of a reference strain for the reordering of contigs is now automated based on the knowledge acquired in the species identification step. Thus, the new pipeline version is 63 times faster than its predecessor in assembling genomes [Figure 2].

For the functional classification of genes, the previous version of the pipeline chooses the first hit between the 10 hits that possess an appropriate protein name. Due to the gradually expanding of the reference database, this strategy is not optimal. Thus, MEGAnnotator2 is provided with pre-processed databases where non-appropriate protein names were previously removed. So, the best hit will automatically represent an orthologous gene with an appropriate protein name. In addition, the novel database is more manageable, and the computing time has been decreased from 60.3 min using MEGAnnotator to 2.9 min in MEGAnnotator2.

Accordingly, the past version of the pipeline was 43 times slower than MEGAnnotator2 in providing the assembled genomes and the annotation of genes, showing an improvement of 20x in the annotation of genes and 63x in the assembly of genomes [Figure 2].

### Performance of the pre-processed RefSeq database of NCBI

In addition to the selection of more efficient software for the execution of each task, one of the major improvements to the pipeline is represented by the pre-processed RefSeq database of NCBI. To select the optimal strategy to assign functional annotation to gene sequences, we employed the genomic repertoire of *Geobacter lovleyi* SZ (CP001089), constituting 3,623 genes, and subsets of the RefSeq database of NCBI. First, RefSeq genes were processed by removing non-informative genes, such as hypothetical proteins, and a collection of unsuitable gene names that may compromise the goodness of the resulting functional classification. Then, RefSeq genes were clustered with CD-HIT using a sequence identity threshold of 90%, 80%, and 70%. Finally, RAPSearch2 and DIAMOND generated databases for taxonomy annotation tests.

The reduction in the size of the database was heavily dependent on the software used and the level of clustering among genes, *i.e.*, from 492.9 to 35.1 GB using RAPSearch2 and from 96.8 to 6.2 GB using DIAMOND [Figure 2I]. Similarly, the speed performance between the two software and the clustered database was significantly lower using DIAMOND (on average twice faster than RAPSearch2), and the RefSeq database builds with a 70% clustering (33 times faster than the RefSeq and 2.8 times faster than clustering at 80%) [Figure 2D].

The resulting functional annotation from both strategies and clustered RefSeq databases does not highlight significant differences [Supplementary Table 4], while classification from the unfiltered RefSeq was superficial due to the imprecise gene classification of the un-processed database. Thus, the software DIAMOND and clustering at 70% by CD-HIT has been selected for their speed advantages and reduced memory usage. This strategy allowed us to build a consistent database for the functional classification of genes constituting a fraction of the RefSeq database (1/80) and achieving the classification of genes 33 times faster. Pre-processed databases will be updated twice a year to guarantee the inclusion of novel genes.

### Benchmark of synthetic datasets

Short- and long-read synthetic datasets were produced from complete genome sequences downloaded from the NCBI repository. In this context, the genome sequence of *Bifidobacterium bifidum* ATCC 29521, *Pseudomonas aeruginosa* ATCC 27853, *Escherichia coli* K-12, *Streptococcus pneumoniae* TIGR4, *Clostridium perfringens* JXJA17, and *Salmonella enterica* MAC15 were chosen, to cover genomes ranging from two to seven Mb [Supplementary Table 5]. The tool wgsim (https://github.com/lh3/wgsim) was used to generate one million synthetic short-read sequences and 150,000 synthetic long-read sequences per genome. Then, the MEGAnnotator2 pipeline was employed to simulate the genome assemblies of each microorganism using a combination of synthetic short- and long-reads. Results highlighted that using long-reads, the integrity of the genomes was higher, allowing the reconstruction of repetitive genome portions that were lost using short-read only, *i.e.*, larger genome sizes and numbers of identified rRNA genes [Supplementary Table 5]. Looking at the execution time of each step of the pipeline, we validate the data previously observed with real samples [Supplementary Table 6]. Hybrid and long-read strategies were more time-consuming, taking double the assembly time with respect to short-read assemblies, as well as the filtering step of long-read sequences [Supplementary Table 6].

Furthermore, the assembly of complex samples was simulated using a limited number of short-read sequences, *i.e.*, 100,000 reads per genome. This synthetic benchmark aimed to test the pipeline if the quality of the sequencing reads were not as good as expected, thus resulting in a few amount of DNA sequences to assembly. In this scenario, the reconstruction of genomes ended whit low average coverage, ranging from 8 to 25, but the integrity of the genomes was maintained, resulting in genome completeness ranging from 96.43% to 99.2% [Supplementary Table 5]. Altogether, the MEGAnnotator2 report showed that the complex genome structure of *Pseudomonas aeruginosa* ATCC 27853 was difficult to assemble, resulting in 663 contigs [Supplementary Table 5].

## CONCLUSIONS

MEGAnnotator2 is a pipeline that manages all the currently existing sequencing formats of modern DNA sequencing systems, including short and long reads. Most of the software associated has been changed to improve the quality of the results and the execution time of the pipeline [Table 1 and Figure 2]. Furthermore, additional features such as read quality filtering, a quality check of DNA and assembled genomes, and metabolic profiling have been added to provide the user with more information and flexibility in the execution of programs. Notably, the execution time from the previous pipeline version has decreased by 43 times, and multiple genomes can be processed in series to avoid wasting time between genome analyses. Furthermore, the pipeline installation does not require additional actions from the user, and the space on the disk of the functional annotation database has been reduced by 80 times. Altogether, MEGAnnotator2 displays all the features needed for the reconstruction of procaryotic and unicellular eukaryotes and can be easily implemented by the user with additional features due to the modulatory architecture of the pipeline.

## DECLARATIONS

### Availability of data and materials

The MEGAnnotator2 pipeline is downloadable at http://probiogenomics.unipr.it/cmu/. The installer file can be retrieved under the “Software & Tools” drop-down menu, section “MEGAnnotator2”, button “Download MEGAnnotator2”. In the same section, the manual can be downloaded using the button “Download Manual”.

### Copyright

© The Author(s) 2023.
